# Association between psychological stress and mandibular condyle structure: an analytical cross-sectional study

**DOI:** 10.1186/s12891-024-07692-8

**Published:** 2024-07-19

**Authors:** Fatemeh Ghasemzadeh, Nazanin Mortazavi, Mysa Mallahi, Mohammad Hadi Gharib, Naser Behnampour, Mohammad Taghi Badeleh, Negar Asgari

**Affiliations:** 1 Dental Research Center, Golestan University of Medical Sciences, Gorgan, Iran; 2 Department of Oral and Maxillofacial Radiology, School of Dentistry, Golestan University of Medical Sciences, Gorgan, Iran; 3 Department of Radiology, School of Medicine, 5th Azar Hospital, Golestan University of Medical Sciences, Gorgan, Iran; 4 Health Management and Social Development Research Center, Golestan University of Medical Sciences, Gorgan, Iran; 5 Department of Psychology, School of Medicine, Golestan University of Medical Sciences, Gorgan, Iran; 6 Department of Microbiology, School of Medicine, Golestan University of Medical Sciences, Gorgan, Iran

**Keywords:** Mandibular Condyle, Stress, Psychological, Temporomandibular Joint, CT scan, X-Ray

## Abstract

**Objective:**

The potential influence of psychological factors on temporomandibular joint disorders has been clinically documented. To date, all research examining the impact of psychological stress on the temporomandibular joint has been conducted on animals. This study aims to explore the relationship between psychological stress and the structure of the human mandibular condyle.

**Methods:**

This cross-sectional study was performed on individuals, who were referred to the radiology division of 5th Azar Hospital for head and neck Computed Tomography (CT) scans. All participants completed a perceived stress questionnaire to determine their level of stress. Bone density and cortical bone thickness were measured as indicators of mandibular condyle structure. Based on multi-slice CT scan data, bone density was calculated in the anterior, middle, and posterior mandibular condyle. The cortical bone thickness was also measured at the anterior and posterior mandibular condyle. Statistical analysis was performed in R 4.0.2 software.

**Results:**

Seventy individuals, aged 18–59 years, participated in this study. The CT scans revealed a decrease in Hounsfield units (HU) and bone mineral density (BMD) in both the anterior and posterior regions. However, in the high-stress group, there was no significant difference in cortical bone thickness in the anterior and posterior regions of the condyle, nor in HU and BMD in the middle region of the condyle. An inverse correlation was observed between BMD and perceived stress in the anterior, middle, and posterior regions of both condyles.

**Conclusion:**

The current findings indicate that recent psychological stress is associated with changes in the structure of the condyle.

## Introduction

The temporomandibular joint (TMJ) is a complex synovial joint composed of a disc, bone, fibrous capsule, synovial fluid, and ligaments. Unlike other joints, the articular surfaces of the TMJ are covered by fibrocartilage rather than hyaline cartilage [1]. This fibrocartilaginous tissue plays a crucial role in absorbing shocks, bearing loads, and providing lubrication. For the TMJ to adapt histomorphologically to mechanical loading, both functionally sound articular cartilage and the subchondral bone beneath it are essential [2, 3]. The Diagnostic Criteria for Temporomandibular Disorders (DC/TMD) serves as a reliable and valid reference standard for the diagnosis of common TMDs in both clinical and research settings. Axis I assessment focuses on assessing physical findings related to TMDs, including pain, functional limitations (such as range of jaw opening), joint sounds, and joint tenderness. Meanwhile, Axis II assessment provides information about the patient’s mental status and quality of life [4].

Evidence suggests that patients with temporomandibular dysfunction (TMD) have higher levels of urinary cortisol and creatinine ratios, indicating elevated levels of emotional stress [5]. Current clinical reports and epidemiological studies have clearly shown that psychological factors play a role in the etiology, persistence, and progression of TMD [6–8]. Additionally, animal models have demonstrated that the TMJ of rodents behaves similarly to the human joint. Consequently, researchers have used animal models in their studies. A recent animal study concluded that biomechanical stress, emotional stress, and estrogen hormones may contribute to TMD [9].

An approach called the communication box method has been recently employed to study physiological responses to psychological stress, this method has been employed to induce ultrastructural changes in the TMJ of rats, which may play a significant role in TMD [10, 11]. In this model, psychological stress in a rat can induce ultrastructural changes in the TMJ, which may play an important role in TMD [12]. Pro-inflammatory cytokines, such as tumor necrosis factor-alpha (TNF-α) and interleukin-1β (IL-1β), contribute to cartilage destruction, which can ultimately result in TMD [13–15]. The application of anti-anxiety medications can provide a reference for the treatment of stress-related TMD [16].

Recent studies have further supported the association between psychological stress and TMJ disorders [17]. For instance, increased biomarkers of stress, such as salivary cortisol, have been found in patients with TMJ pain, demonstrating the link between stress and TMJ disorders [18]. Additionally, a systematic review confirmed the association between anxiety and TMD, highlighting the significant role of psychological factors in TMD [17].

Today, high-resolution computed tomography (CT) scans are used to quantify bone structure and evaluate bone strength [19, 20]. The use of high-resolution imaging in laboratory studies has been gradually incorporated into human clinical trials [21]. The present study aimed to determine whether psychological stress impacts the mandibular condyle structure in healthy humans and individuals with psychological stress, using multi-slice three-dimensional quantitative CT scans.

## Materials and methods

This study was performed on individuals who were referred to the Radiology Division of 5th Azar Hospital, affiliated with Golestan University of Medical Sciences (Gorgan, Iran), by their physicians for head and neck CT scans as part of their medical management. All participants signed an informed consent form to participate in the study.

### Inclusion criteria

The inclusion criteria in this study were as follows: (1) no history of mandibular trauma; (2) no clicking; (3) no limitations in opening or lateral movements of the mandible (≥ 4 cm mouth opening and ≥ 1 cm lateral movement); (4) absence of more than four posterior teeth unilaterally or bilaterally; (5) no systemic disease affecting the TMJ (e.g., rheumatoid arthritis, scleroderma, lupus, sarcoidosis, psoriasis, or Behcet’s disease); (6) no parafunctions (e.g., bruxism and clenching); and (7) age of 18 years or above [22].

### Sample size

There was no similar human study to the present study in the literature, so the sample size was calculated based on preliminary results from an eight-subject pilot study. The sample size was determined to be 63 individuals, using the correlation coefficient formula witα = 0.05, β = 0.2, and a correlation coefficient of 0.35.

$$\:n=\left[\frac{{\left({Z}_{1-\alpha\:}+{Z}_{1-\beta\:}\right)}^{2}}{{\left(\frac{1}{2}\text{ln}\frac{1+r}{1-r}\right)}^{2}}\right]+3$$

### Perceived stress scale

The 14-item Perceived Stress Scale (PSS-14) was administered to assess the level of stress in all the participants. PSS-14 was designed by Cohen and colleagues in 1983. Since radiography may cause stress for individuals, this scale was completed on days other than the radiography day.

### Data collection and data analysis

In the present study, a Siemens Multi-slice CT scanner was used for radiography (110 kV, 40 mA, integration time, 0.6–1 s). The pixel size of images was set at 512 × 512, and the slice thickness was set at 0.75 mm. The OsiriX MD software was used to study images of the largest axial slice of the condyle and to calculate the Hounsfield unit (HU) in the anterior, middle, and posterior aspects of the condyle, as well as the cortical thickness in the anterior and posterior aspects of the condyle.

In a cadaver mandible study by Homolka et al. [23], the CT numbers were converted to local bone mineral density (BMD) values, based on the assumption of a linear relationship (BMD = a×HU + b.

where a and b are the calibration coefficients). The calibration coefficients for the patients were measured to be a = 0.804 ± 0.06.

and b = 5.2 ± 4.2.

Overall, by calculating BMD along with cortical thickness, a comprehensive description of the bone can be obtained. Two methods were used to determine the association between psychological stress and mandibular condyle structure. In addition to calculating the correlation coefficient between the psychological stress score and mandibular condyle structure, the subjects were divided based on the psychological stress score, considering a score of ≤ 18.

as the low-stress group and a score of ≥ 38.

as the high-stress group. The Shapiro-Wilk test was used to evaluate the normality of data distribution. Welch’s t-test was used to compare the mean HU and BMD indices in the anterior, middle, and posterior parts of the condylar head, as well as the cortical bone thickness in the anterior and posterior areas. The Mann-Whitney U test was also used for indices of abnormal distribution.

## Results

A total of 70 individuals (36 men and 34 women), aged 18–59 years, participated in this study. The student’s t-test and Chi-square test revealed no statistically significant differences between the two groups regarding age and sex. The mean values of HU and BMD for the anterior part of the right condyle were 168.38 and 135.37 units lower in the high-stress group compared to the low-stress group, respectively. The independent t-test showed statistically significant differences between the means of the groups, indicating that high-stress individuals had lower values of these indices.

For the anterior part of the left condyle, the mean values of HU and BMD were 177.15 and 142.42 units lower in the high-stress group compared to the low-stress group, respectively. These differences were statistically significant according to the independent t-test, suggesting that individuals with high levels of stress had lower values of these indices. Additionally, the mean values of HU and BMD for the middle part of the right condyle were 39.43 and 81.7 units lower in the high-stress group compared to the low-stress group, respectively. However, the Mann-Whitney U test indicated that these differences were not statistically significant.

For the middle portion of the left condyle, the mean values of HU and BMD were 22.26 and 19.5 units lower in the high-stress group compared to the low-stress group, respectively. The independent t-test did not indicate a significant difference between the groups, and the high and low-stress participants had similar mean values of these indices. Moreover, the mean values of HU and BMD in the posterior part of the right condyle were 98.5 and 79.2 units lower in the high-stress group compared to the low-stress group, respectively. The independent t-test showed no significant difference between these groups, indicating that the mean values of these indices were not significantly different between high-stress and low-stress groups.

For the posterior part of the left condyle, the mean values of HU and BMD were 106.59 and 85.69 units lower in the high-stress group compared to the low-stress group, respectively. The Mann-Whitney U test showed an approximately significant level, suggesting that the mean values of these indices were relatively lower in individuals with high-stress levels compared to those with low levels of stress.

The mean cortical bone thickness of the anterior part of both right and left condyles was 0.07 and 0.08 units higher in the high-stress group compared to the low-stress group, respectively. However, the Mann-Whitney U test revealed no significant difference between the groups, indicating that this index was similar in individuals with high and low levels of stress. Additionally, the mean cortical bone thickness in the posterior parts of the right and left condyles was 0.03 and 0.04 units higher in the high-stress group compared to the low-stress group, respectively. The independent t-test revealed that the difference was not statistically significant, indicating that the mean values of this index were similar in both groups with high and low levels of stress (Table 1).

**Table 1:** Mean (SD) of bone structure in both high-stress and low-stress groups

Index	Groups	Condyle Area					
		Anterior Right Condyle	Anterior Left Condyle	Posterior Right Condyle	Posterior Left Condyle	Middle Right Condyle	Middle Left Condyle
Hu	High-stress	1412.2 (234.5)	1361.7 (228.1)	1053.7 (309.72)	958.68 (363.26)	328.45 (92.7)	335.91 (79.135)
	Low-stress	1580.6 (264.8)	1538.9 (238.6)	1152.23 (279.40)	1065.27 (274.13)	367.88 (113.2)	360.17 (106.93)
	P Value	0.013 ^1^	0.005 ^1^ *	0.190 ^1^	0.065 ^2^	0.400 ^2^	0.346 ^1^
BMD	High-stress	1140.6 (188.5)	1100.06 (183.4)	852.39 (249.01)	775.98 (292.06)	269.2 (74.54)	275.27 (63.6)
	Low-stress	1276.0 (212.9)	1242.48 (191.8)	931.59 (224.63)	861.67 (220.40)	300.9 (91.0)	294.7 (85.97)
	P Value	0.013 ^1^	0.005 ^1^ *	0.190 ^1^	0.065 ^2^	0.400 ^2^	0.346 ^1^
Cortical bone thickness	High-stress	1.06 (0.30)	1.08 (0.29)	0.98 (0.18)	1.00 (0.18)		
	Low-stress	0.99 (0.15)	1.00 (0.17)	0.95 (0.14)	0.96 (0.14)		
	P Value	0.510 ^2^	0.229 ^2^	0.429 ^1^	0.396 ^1^		

^1^ Independent T-test; ^2^ Mann-Whitney U test

As BMD is calculated based on HU indices, their P Values will be equal.

**Fig. 1:**
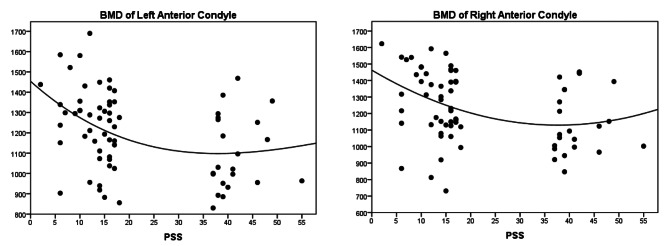
Curve fitting for BMD of anterior right and left condyles and PSS

To determine the association of BMD and cortical bone thickness with perceived stress, Pearson’s correlation coefficient (for normally distributed variables) and Spearman’s correlation coefficient (for variables without a normal distribution) were measured (Table 2; Fig. 1).

**Table 2:** Correlation (P-value) of condylar structure indices and PSS

Index	Condyle Area	Anterior	Posterior	Middle
BMD	Right	-0.329 (0.005) *	-0.128 (0.293)	-0.133 ^2^ (0.247)
	Left	-0.358 (0.002) *	-0.183 ^2^ (0.130)	-0.148 (0.222)
Cortical bone thickness	Right	0.060 ^2^ (0.624)	0.073 (0.550)	-
	Left	0.119 ^2^ (0.325)	0.062 (0.611)	

^1^ Pearson correlation coefficient; ^2^ Spearman’s rank correlation coefficient

The results indicate that BMD is inversely related to perceived stress in the anterior, middle, and posterior regions of both condyles. This relationship is significant in the anterior region of both condyles (p-Value < 0.005). The HU, BMD, and cortical bone thickness of the left and right condyles were calculated for the high-stress and low-stress groups. However, no significant difference was found between the left and right condyles in any of the condylar areas. On the other hand, the HU and BMD of the posterior part of the right condyle were 189.9 and 152.7 units higher in women compared to men, respectively, and the difference was statistically significant.

Additionally, the relationship between age and condylar structure indices was determined using Pearson’s correlation or Spearman’s correlation test. The bone density of the anterior and posterior parts of the right condyle had a significant inverse relationship with age; a third-grade model showed a significant relationship between the bone density of the anterior right condyle and age (p-Value = 0.034). Besides, the cortical bone thickness of the anterior surface of the left condyle was directly related to age (p-Value = 0.031). In older subjects, the cortical bone thickness of the anterior part of the left condyle was greater, while the bone density of the anterior and posterior parts of the right condyle was lower.

The bone density of the middle and posterior areas of the left condyle had an inverse correlation with age, which was almost significant. The cortical bone thickness of the anterior and posterior surfaces of the right condyle, as well as the bone density of the middle part of the right condyle, had an inverse correlation with age, which was not statistically significant. Besides, the cortical thickness of the anterior and posterior parts of the left condyle had a direct correlation with age; nonetheless, it was not statistically significant (Table 3).

**Table 3:** Correlation (p-value) of condylar structure indices and age

Index	Condyle Area	Anterior	Posterior	Middle
BMD	Right	-0.306 (0.010) *	-0.318 (0.007) *	-0.138 ^2^ (0.254)
	Left	-0.164 (0.174)	-0.233 ^2^ (0.053)	-0.207 (0.086)
Cortical bone thickness	Right	-0.019 ^2^ (0.878)	-0.030 (0.804)	-
	Left	0.259 ^2^ (0.031) *	0.132 (0.275)	

^1^ Pearson correlation coefficient; ^2^ Spearman’s rank correlation coefficient

## Discussion

The TMJ experiences morphological changes as individuals age, impacting its function. Older age and male sex correlate with reduced BMD in specific condylar regions, while cortical bone thickness may increase elsewhere. Additionally, psychological stress plays a role in TMD symptoms and joint health. High stress levels are associated with decreased BMD in the anterior part of the condyle [24]. In addition to psychological stress, several factors—such as age, physical activity levels, vitamin D intake, hormonal changes, and genetic predisposition—collectively influence bone density. These elements impact the bone remodeling process, ultimately affecting BMD and overall bone health. When assessing changes in the mandibular condyle structure, considering these factors is crucial [25].

In the present study, the association between psychological stress and mandibular condyle structure was investigated. The HU and bone density of the posterior part of the left condyle are relatively lower in individuals with high levels of stress compared to those with low levels of stress. However, the mean HU of the posterior aspect of the right condyle and the cortical bone thickness in the posterior part of the right and left condyles were not significantly different between high-stress and low-stress groups.

In their study, Li et al. [3] investigated the left condyle of rats exposed to psychological stress. They found that stress led to a reduction in bone volume fraction (BV/TV) and trabecular thickness, along with an increase in bone surface density (BS/BV) and trabecular separation in the posterior part of the subchondral bone during the fifth week (with corresponding p-values of 0.041, 0.030, 0.025, and 0.043, respectively). These results align with our current study, which also observed similar structural changes in the posterior part of the left condyle.

Interestingly, in individuals with high stress levels, the indices of HU and bone density in the middle part of both right and left condyles did not significantly differ from those in individuals with low stress levels (p-value = 0.05 for all). This finding is consistent with Li et al.‘s study, where no significant difference was observed in the microstructure indices of the middle parts of subchondral bones (p-value > 0.05).

In our recent study, we observed a significant difference between the high-stress and low-stress groups in terms of bone density within the anterior parts of both the right and left condyles (with p-values of 0.013 and 0.005, respectively). However, Li et al.‘s study did not find any significant difference between the low-stress and study groups regarding the microstructure indices of the anterior part of the subchondral bone (*p* > 0.05 for all). This discrepancy in findings may be attributed to variations in TMJ structure between humans and mice, as well as differences in their mastication forces.

Both genetics and environmental factors play pivotal roles in bone density. In fact, genetics is considered the most significant factor, accounting for 60–80% of bone density variation [26]. Our study investigated the effects of perceived stress—an environmental factor—on bone structural indices. We found an inverse correlation between perceived stress and BMD in the anterior part of the mandibular condyle. However, no significant relationship was observed between perceived stress and BMD in the middle and posterior parts of the condyle, nor between perceived stress and cortical bone thickness in the anterior and posterior regions.

Another noteworthy finding in our study was the comparison of structural aspects between the right and left condyles in the high-stress and low-stress groups. Both groups exhibited higher mean values of HU and BMD in the posterior aspect of the right condyle compared to the left condyle (*p* = 0.049 and *p* = 0.008, respectively). In the low-stress group, the mean differences between the anterior aspect of the right condyle (HU and BMD) and the left condyle were relatively significant, with the mean values of the right condyle being higher (*p* = 0.056). These structural differences may be attributed to unilateral chewing habits, unilateral tooth loss, and dental sensitivities. Individuals with a unilateral chewing habit are more likely to develop TMD symptoms [27]. Future studies should consider unilateral chewing habits as a potential confounding factor.

Interestingly, the mean values of HU and BMD did not significantly differ in the anterior parts of the right and left condyles within the high-stress group. Neither the low-stress group nor the high-stress group showed significant differences in the mean HU and BMD values in the middle areas or the mean cortical thickness of the anterior and posterior aspects of the right and left condyles. Given the limited studies examining the relationship between cone beam computed tomography (CBCT) density and perceived stress (primarily in rats), there remains insufficient information regarding mandibular condyle changes in individuals with high stress levels. Furthermore, the correlation of perceived stress with structural indices in human condylar bone has not been thoroughly investigated.

In our study, we also explored the relationship between sex and condylar bone indices. Women exhibited higher HU and BMD values in the posterior aspect of the right condyle (*p* = 0.005). Additionally, compared to men, women had relatively higher HU and BMD values in the anterior part of the right condyle (*p* = 0.053). These findings align with a study by Schoenau et al.‚ [28] which reported higher cortical BMD in women after puberty and before menopause. Conversely, Salimov et al., in their investigation of trabecular bone density and implant stability indices, found that men had higher BMD, possibly due to hormonal differences, greater bone mass in men, and varying aging patterns between genders [29].

In our recent study, we observed that bone density in the anterior and posterior parts of the right condyle was lower in older subjects, while cortical bone thickness was greater in the anterior part of the left condyle. Additionally, the BMD of the medial and posterior parts of the left condyle was relatively lower in older individuals. We also found inverse relationships between age and BMD in the middle part of the right condyle and the cortical bone thickness of the anterior and posterior aspects of the right condyle; however, these correlations were not statistically significant.

Interestingly, Riggs et al. reported an inverse linear correlation between age and BMD in their study [30]. Similarly, Do Lee et al. identified age as the most important clinical index for predicting bone density and osteoporosis [31]. However, Salimov et al. found that bone density increased with age [29]. This discrepancy might be attributed to increased bone resorption in older age, resulting in more basal bone remaining.

The present study presents several limitations. First, the cross-sectional design prevents causality between stress and changes in condyle structure. Second, reliance on self-reported stress levels may introduce bias, as they may not accurately reflect actual stress. Third, the sample size, although calculated based on a pilot study, may not be representative of the general population. Additionally, the study did not take into account other factors such as diet, genetics or lifestyle that can affect bone density and structure. Finally, the findings are based on a specific population, which may limit the generalizability of the results to other groups or settings. Despite these limitations, the study is original in its approach, as it is one of the first to explore the impact of psychological stress on the human mandibular condyle using high-resolution CT scans, providing valuable insights into the potential link between stress and temporomandibular joint disorders.

## Conclusion

Based on the present results, individuals with high-stress levels had lower BMDs in the anterior aspect of the condyle. The results indicated that older age and male sex were associated with a decrease in the BMD of the anterior and posterior aspects of the condyles and an increase in cortical bone thickness. Overall, the current results suggest an association between psychological stress and changes in the mandibular condyle structure.

## Data Availability

The datasets generated and/or analyzed during the current study are not publicly available due to confidentiality of information but are available from the corresponding author on reasonable request.
